# Supplementary material for: Towards Unbiased Evaluation of Ionization Performance in LC-HRMS Metabolomics Method Development
Source: Metabolites. 2022 May 10;12(5):426. doi: 10.3390/metabo12050426 (PMC9144264; doi:10.3390/metabo12050426)
Supplement: Supplementary file 1 [file metabolites-12-00426-s001.zip › metabolites-1646579-supplementary.pdf]

Supplementary Materials

for the manuscript:

Towards unbiased evaluation of ionization performance in LC-HRMS metabolomics method development

Carsten Jaeger and Jan Lisec

Bundesanstalt für Materialforschung und -prüfung (BAM), Department 1 Analytical Chemistry; Reference Materials, Richard-Willstätter-Straße 11, 12489 Berlin, Germany

- submitted to *Metabolites* -

Supplementary Figures

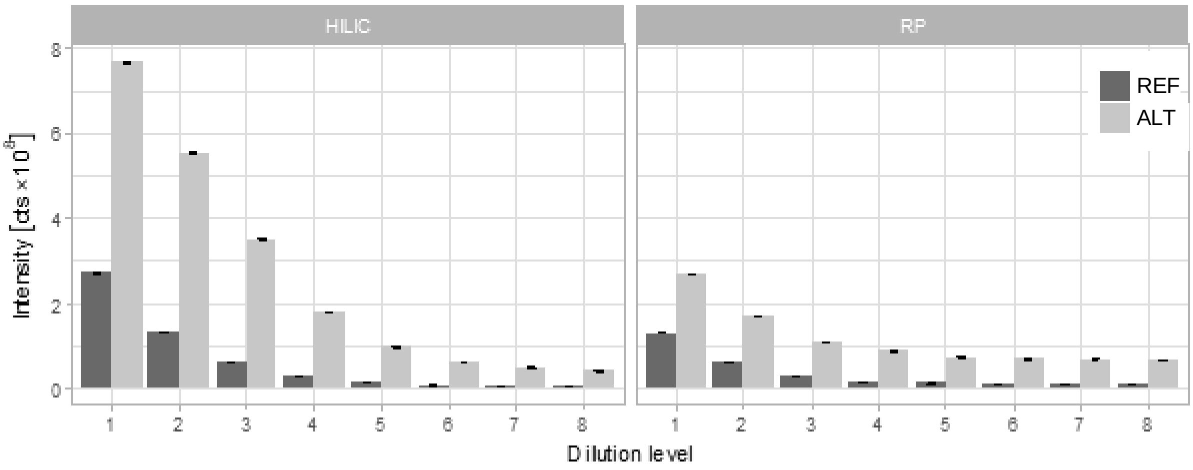

Figure S1. Comparison of total intensity signals measured for a biological test sample at different concentration levels using the reference (REF) and alternative (ALT) instrumental setup, respectively. Results show a large offset in sensitivity between the setups, leading to difficulties in directly comparing measurements results. Evaluation was separately carried for two chromatography modes investigated (HILIC, RPC). Bars represent mean summed base peak intensites (±SD) of four repeated injections.

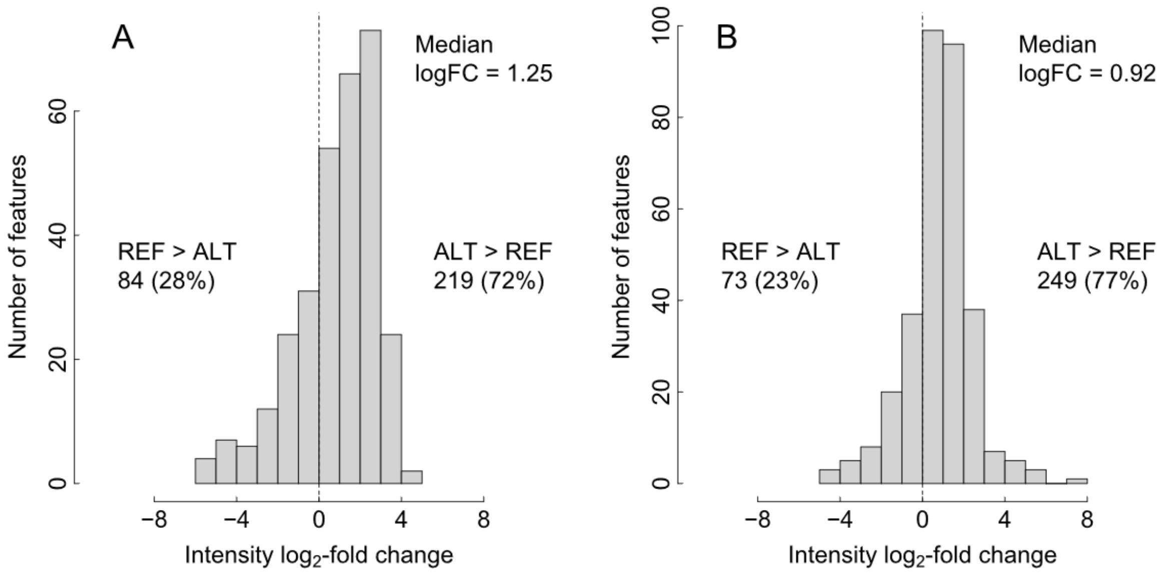

Figure S2. Evaluation of selectivity based on targeted data. A library of metabolite standards was analyzed by LC-HRMS using two instrumental setups (REF, ALT) and $\log_2$-fold changes in intensities were calculated for protonated and deprotonated molecular ion species ($[M+H]^+$, $[M-H]^-$), respectively. Histograms show data for the HILIC (A) and RPC (B) methods, respectively. Percentages of compounds with enhanced intensity in one of the setups are indicated beside the histograms; these refer to the total number of compounds with acceptable (symmetric) peak shapes within each method (303 for HILIC and 322 for RPC). See Figure 2 for the corresponding nontargeted evaluation.

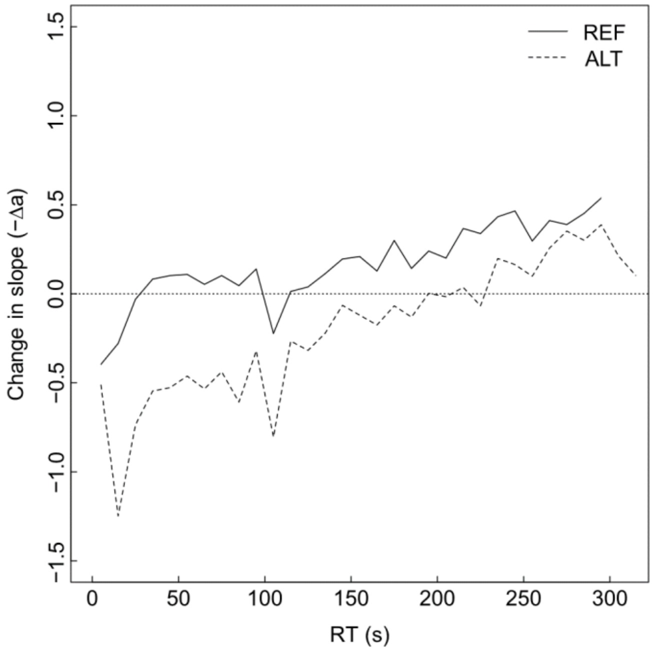

Figure S3. Evaluation of ion suppression based on nontargeted data for the complementary reversed phase (RPC) subset. See legend of Figure 3 for an explanation.

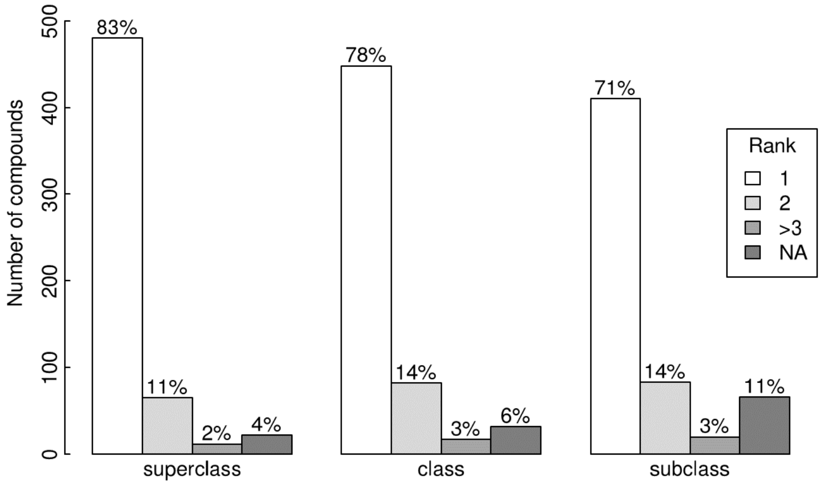

Figure S4. Test of chemical classification based on targeted data. Spectra of known metabolite standards (n = 578) were annotated with MS-FINDER and classified with ClassyFire. The percentage of correct classification at the respective ontological level ("superclass", "class", "subclass") is given. "Rank" refers to the scored metabolite IDs from MS-FINDER with 'NA' designating that no ID was in the correct class.

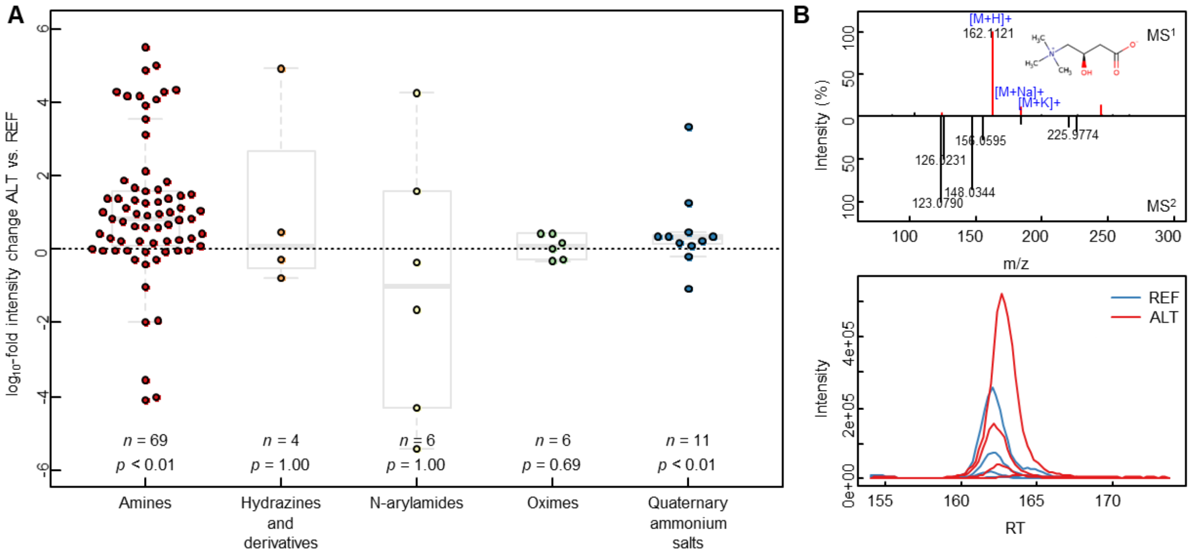

Figure S5. Exemplary changes in selectivity as detected by chemical classification of nontargeted data. (A) Change in analytical response ALT vs. REF setups for individual metabolites, color-coded by chemical subclass (superclass "Organic nitrogen compounds"). Metabolite counts and p values of a binomial test are given; p values < 0.05 indicate significant changes. (B) Pseudospectrum and EICs for L-carnitine, as a sample quaternary ammonium compound.

Supplementary Tables

Table S1. Chemical classification of the nontargeted dataset. Deconvoluted spectra were identified with MS-FINDER followed by chemical classification with ClassyFire. Columns summarize numbers *(n)* and feature intensities *(I)* summed by chemical class. REF/ALT: reference/alternative analytical setup; HILIC/RPC: chromatography method used.

**Chromatography method**	**HILIC**										**RPC**									
**Instrumental setup**	**REF**					**ALT**					**REF**					**ALT**				
**Compound class**	*n*	*n* (%)	*I* (×10^6)	log$_{10}$-*I*	log$_{10}$-*I* (%)	*n*	*n* (%)	*I* (×10^6)	log$_{10}$-*I*	log$_{10}$-*I* (%)	*n*	*n* (%)	*I* (×10^6)	log$_{10}$-*I*	log$_{10}$-*I* (%)	*n*	*n* (%)	*I* (×10^6)	log$_{10}$-*I*	log$_{10}$-*I* (%)
Alkaloids and derivatives	6	1.1	0.9	23.8	1.1	9	2.8	1	38.3	2.7	7	1.6	0.2	28.6	1.6	5	1.5	0.2	22.2	1.6
Benzenoids	61	11.3	12.5	262.1	11.7	40	12.2	18.9	183.1	12.9	53	12.4	2.7	219.6	12.6	32	9.6	1.4	125.9	9.1
Homogeneous non-metal compounds	1	0.2	0	0	0	1	0.3	0	0	0	0	0	0	0	0	0	0	0	0	0
Hydrocarbons	3	0.6	0	10.2	0.5	0	0	0	0	0	3	0.7	0.1	10.3	0.6	0	0	0	0	0
Lignans, neolignans and related compounds	1	0.2	0.1	5	0.2	0	0	0	0	0	2	0.5	0.2	8.5	0.5	0	0	0	0	0
Lipids and lipid-like molecules	38	7	7.6	168.2	7.5	21	6.4	6.3	91.7	6.5	74	17.2	1.5	280.9	16.1	39	11.7	5	166.1	12
Nucleosides, nucleotides, and analogues	16	3	1.1	65.8	2.9	4	1.2	1.6	20.5	1.4	6	1.4	0.1	23.6	1.4	10	3	0.3	42	3
Organic 1,3-dipolar compounds	3	0.6	0.1	12.5	0.6	0	0	0	0	0	1	0.2	0	3.6	0.2	0	0	0	0	0
Organic acids and derivatives	196	36.2	36	844.1	37.6	93	28.4	37.8	394.6	27.9	109	25.4	11	456.2	26.1	97	29.1	6.5	407.2	29.4
Organic nitrogen compounds	27	5	4.5	118.8	5.3	34	10.4	7.3	143.5	10.1	9	2.1	0.2	30.3	1.7	37	11.1	1.3	145	10.5
Organic oxygen compounds	64	11.8	3.8	247.3	11	29	8.9	6.2	125.6	8.9	52	12.1	2.9	211.6	12.1	50	15	2.2	211.8	15.3
Organoheterocyclic compounds	121	22.4	20.4	470.9	21	87	26.6	20.4	386.2	27.3	93	21.7	11	397.1	22.7	56	16.8	2.4	235.1	17
Organosulfur compounds	1	0.2	0	4.1	0.2	3	0.9	0.2	9.1	0.6	3	0.7	0.3	13.4	0.8	1	0.3	0	4.1	0.3
Phenylpropanoids and polyketides	3	0.6	0.4	12.8	0.6	6	1.8	0.7	23.2	1.6	17	4	0.4	65.5	3.7	6	1.8	0.1	25.1	1.8
Sum	541	100	87	2246	100	327	100	100	1416	100	429	100	31	1749	100	333	100	19	1385	100

Table S2. Composition of Pharmaceutical Mix 17 (Neochema, Germany) used as internal standard and for ion suppression assay.

Substance	CAS no.	Stock concentration (µg mL^{-1})	Final concentration (internal standard; µg mL^{-1})	Working concentration (ion suppression assay; µg mL^{-1})
Atenolol	29122-68-7	10	0.2	0.2
Bezafibrate	41859-67-0	10	0.2	0.2
Bisoprolol	66722-44-9	10	0.2	0.2
Carbamazepine	298-46-4	10	0.2	0.2
Clofibric acid	882-09-7	10	0.2	0.2
Diclofenac (free acid)	15307-86-5	10	0.2	0.2
Ibuprofen	15687-27-1	10	0.2	0.2
Ketoprofen	22071-15-4	10	0.2	0.2
Metoprolol	51384-51-1	10	0.2	0.2
Nadolol	42200-33-9	10	0.2	0.2
Naproxen	22204-53-1	10	0.2	0.2
Phenazone	60-80-0	10	0.2	0.2
Propranolol-HCl	318-98-9	10	0.2	0.2
Propyphenazone	479-92-5	10	0.2	0.2
Salbutamol	18559-94-9	10	0.2	0.2
Sotalol-HCl	959-24-0	10	0.2	0.2
Terbutaline hemisulfate	23031-32-5	10	0.2	0.2
